# A Rare Cause of Retropharyngeal Abscess: Cervical Pott's Disease

**DOI:** 10.4269/ajtmh.14-0460

**Published:** 2015-05-06

**Authors:** Awad Ali M. Alawad, Amr Fathi M. Khalifa

**Affiliations:** Surgery, University of Medical Sciences and Technology, Khartoum, Sudan; Otolaryngology, Military Hospital, Khartoum, Sudan

**Figure 1.**
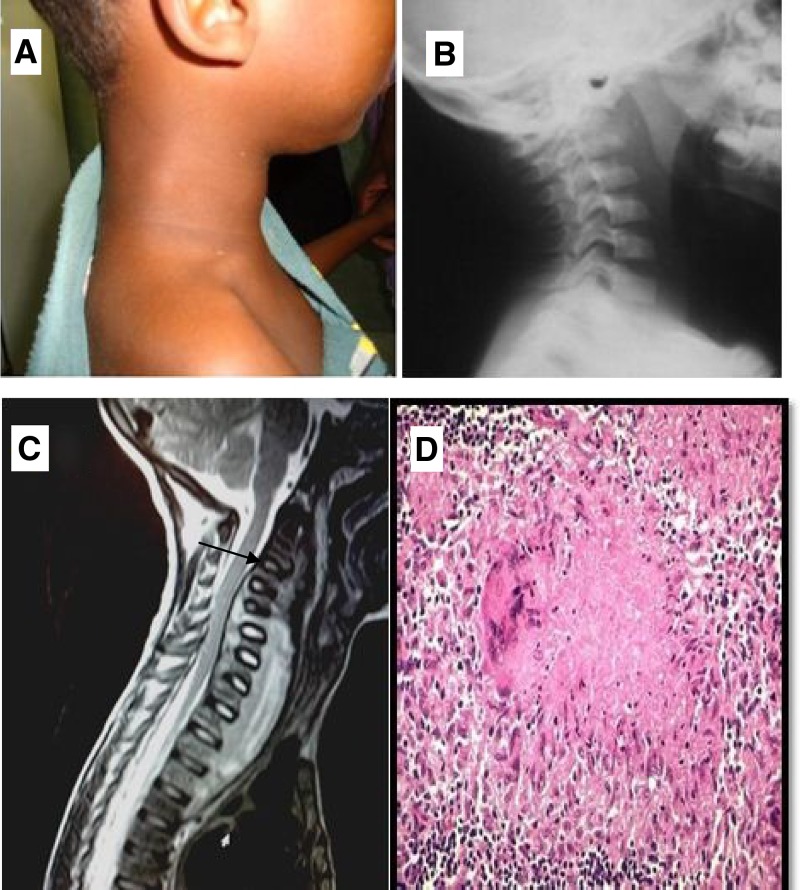
(**A**) Neck swelling and stiffness observed on the right side. (**B**) A lateral cervical X-ray showed widening of the retropharyngeal space. (**C**) MRI revealed a decrease in vertebral body height, irregular margins, and a decrease in intravertebral disk space at C3–C4 and destructed C1 vertebra (marked by the black arrow). (**D**) Photomicrograph revealed caseating granuloma with central necrosis, lymphocytes, and giant cells, which are consistent with tuberculosis.

A 6-year-old boy presented with 4 months of right neck pain that, along with increasing swelling over the previous 15 days, was associated with fever, night sweats, and dysphagia. Physical examination showed diffuse right neck swelling (Figure 1A). Serological tests for human immunodeficiency virus (HIV) were negative. A lateral cervical spine X-ray revealed widening of the retropharyngeal space (Figure 1B). Magnetic resonance imaging (MRI) revealed a hyperintense pre-vertebral collection (Figure 1C). A percutaneous fine-needle aspiration decompressed the collection. Microscopic examination of the material obtained showed acid fast bacilli and granulomatous inflammation (Figure 1D). Administration of antituberculous drugs (isoniazid, rifampicin, and pyrazinamide) led to rapid improvement (disappearance of visible swelling at 4 weeks), especially the ability to eat normally. Six months of treatment was completed. Retropharyngeal abscess caused by cervical Pott's Disease is rare and should be suspected with a destructive spine lesion with associated findings in the appropriate setting.^1^,^2^
